# Evaluation of camera-based freehand SPECT in preoperative sentinel lymph node mapping for melanoma patients

**DOI:** 10.1186/s13550-020-00729-8

**Published:** 2020-11-11

**Authors:** Annie K. Kogler, Andrew M. Polemi, Surabhi Nair, Stanislaw Majewski, Lynn T. Dengel, Craig L. Slingluff, Brian Kross, S. J. Lee, J. E. McKisson, John McKisson, Andrew G. Weisenberger, Benjamin L. Welch, Thomas Wendler, Philipp Matthies, Joerg Traub, Michael Witt, Mark B. Williams

**Affiliations:** 1 Department of Physics, University of Virginia, Charlottesville, VA USA; 2 Department of Biomedical Engineering, University of Virginia, Charlottesville, VA USA; 3 Department of Radiology and Medical Imaging, University of Virginia, Charlottesville, VA USA; 4 Department of Surgery, University of Virginia, Charlottesville, VA USA; 5 Thomas Jefferson National Accelerator Facility, Newport News, VA USA; 6 Dilon Diagnostics, Newport News, VA USA; 7 SurgicEye, GmbH, Munich, Germany

**Keywords:** Image-guided intervention, Freehand SPECT, Sentinel lymph node, Silicon photomultiplier, Handheld gamma camera

## Abstract

**Background:**

Assessment of lymphatic status via sentinel lymph node (SLN) biopsy is an integral and crucial part of melanoma surgical oncology. The most common technique for sentinel node mapping is preoperative planar scintigraphy of an injected gamma-emitting lymphatic tracer followed by intraoperative node localization using a non-imaging gamma probe with auditory feedback. In recent years, intraoperative visualization of SLNs in 3D has become possible by coupling the probe to an external system capable of tracking its location and orientation as it is read out, thereby enabling computation of the 3D distribution of the tracer (freehand SPECT). In this project, the non-imaging probe of the fhSPECT system was replaced by a unique handheld gamma camera containing an array of sodium iodide crystals optically coupled to an array of silicon photomultipliers (SiPMs). A feasibility study was performed in which preoperative SLN mapping was performed using camera fhSPECT and the number of detected nodes was compared to that visualized by lymphoscintigraphy, probe fhSPECT, and to the number ultimately excised under non-imaging probe guidance.

**Results:**

Among five subjects, SLNs were detected in nine lymphatic basins, with one to five SLNs detected per basin. A basin-by-basin comparison showed that the number of SLNs detected using camera fhSPECT exceeded that using lymphoscintigraphy and probe fhSPECT in seven of nine basins and five of five basins, respectively. (Probe fhSPECT scans were not performed for four basins.) It exceeded the number excised under non-imaging probe guidance for seven of nine basins and equaled the number excised for the other two basins.

**Conclusions:**

Freehand SPECT using a prototype SiPM-based gamma camera demonstrates high sensitivity for detection of SLNs in a preoperative setting. Camera fhSPECT is a potential means for efficiently obtaining real-time 3D activity distribution maps in applications such as image-guided percutaneous biopsy, and surgical SLN biopsy or radioguided tumor excision.

## Background

### Sentinel lymph node mapping

Sentinel lymph node biopsy (SLNB) is a widely adopted technique for assessment of lymph node status in cancer patients. Several hours prior to surgery, a subcutaneously injected radioisotope-labeled colloidal tracer, and sometimes a blue dye, is injected in the proximity of the tumor(s). The radiotracer particles drain via the lymphatic system and are trapped in nearby lymph nodes. During surgery, lymph nodes with the highest tracer accumulation (defined as sentinel nodes) are excised and histological node status analysis is performed immediately. If no malignant cells are found, no further nodes are removed, thereby minimizing postsurgical lymphadenectomy-related morbidity.

In current practice, initial visualization of general SLN location is provided by lymphoscintigraphy, in which a large, general-purpose gamma camera acquires planar scintigraphic images immediately following radiotracer drainage. During surgery, a non-imaging handheld gamma probe, providing variable-pitch audio feedback to the surgeon, is used for SLN localization, excision, and ex vivo activity measurement. SLNB is a routine component of surgical management and staging of patients with clinical stage IB and II melanoma, where the pathologic status of the SLN is the most important prognostic factor [1]. However, in a meta-analysis of more than 25,000 patients [2] using the combination of preoperative lymphoscintigraphy and intraoperative gamma probe, node localization false negative rates averaged 12.5%. In cases where the cause for a false negative is able to be identified, 44–50% have been ascribed to failure of radiologic or surgical identification of the sentinel lymph node [3].

Preoperative whole-body SPECT-CT provides a 3D imaging alternative to 2D lymphoscintigraphy in SLNB, with advantages that include more accurate anatomical localization and improved ability to visualize nodes near the injection site [4, 5]. It has been effectively utilized in numerous applications of SLNB, including melanoma, breast cancer, and head and neck cancer [6].

The declipse®SPECT system, also known as freehand SPECT (fhSPECT), was developed by SurgicEye (Munich, Germany) to provide preoperative and intraoperative 3D imaging of radiotracers [7, 8]. As originally developed and marketed, the system uses an overhead infrared imaging system to track the position and orientation in space of a non-imaging probe as it is raster-scanned above the patient surface. Count rate data from the probe’s single detector are streamed to a fast reconstruction algorithm to generate a 3D (fhSPECT) image of the radiotracer distribution that is superimposed upon a live visible light video image of the patient. For 3D localization of SLNs, fhSPECT provides an alternative to preoperative whole-body SPECT and offers a potential means for overcoming several of its drawbacks. These include the inability to update the tracer image immediately prior to or during surgery and thereby dynamically adjust for changes in patient positioning, or to reimage following node excision to assess residual activity. SPECT-CT is also expensive, has limited access at many institutions performing SLNB, and introduces additional radiation dose (from the CT scan). There is evidence that fhSPECT imaging is comparable to SPECT-CT in detection of SLNs. In a study including 15 oral cancer patients, preoperative lymphoscintigraphy and SPECT-CT were followed by intraoperative probe fhSPECT, with surgeons initially blinded to all preoperative imaging. Of the 144 sentinel nodes excised, 95 were identified by lymphoscintigraphy, 122 by SPECT-CT, and 125 by fhSPECT [9].

Freehand SPECT has been successfully used for SLN mapping in a variety of cancers including breast [10, 11] and melanoma [12–15] and has demonstrated identification of a higher number of SLNs compared to conventional gamma probe detection [10]. It has also been successfully employed in localization of preoperatively placed radioactive tumor markers such as the ^125^I seeds used in breast cancer surgery [16]. Fitted with a laparoscopic extension for the gamma probe, declipse®SPECT has also been evaluated for guidance in excision of pulmonary nodules preoperatively marked using [^99m^Tc]macroaggregated albumin using CT guidance [17, 18].

Small field-of-view gamma cameras, typically utilized in 2D scintigraphy mode and mounted on a mobile gantry arm, provide a means to potentially reduce the number of false negatives in SLNB compared to the combination of preoperative lymphoscintigraphy and intraoperative use of a non-imaging probe [19–24]. Such compact imaging devices can be used in the preoperative surgical suite immediately prior to surgery to obtain updated images to guide SLNB planning and can acquire updatable, real-time two-dimensional (2D) images of SLN location in the operating room. In addition to SLNB, mobile gamma cameras have also been evaluated for use in radiomarker localization in procedures such as radioguided occult lesion localization and radioguided seed localization [25, 26].

Recently, the replacement in fhSPECT of the non-imaging gamma probe with a handheld imaging gamma camera has been explored. The potential advantages of fhSPECT using an imaging camera (referred to here as ‘camera fhSPECT’) instead of using a non-imaging gamma probe (referred to as ‘probe SPECT’) include a much larger detection surface area, the simultaneous use of multiple gamma-sensitive detector elements, higher spatial resolution, and more complete tomographic sampling. Engelen et al. have described the evaluation of the combination of declipse®SPECT and a commercially available cadmium zinc telluride (CZT)-based mobile gamma camera (CrystalCam from Crystal Photonics, Berlin, Germany) [27].

In this project, an investigational camera fhSPECT system was developed in which the non-imaging probe was replaced by a unique silicon photomultiplier (SiPM)-based handheld gamma camera. The main goals were to increase the active field of view (FOV) and the completeness of SPECT sampling, while maintaining some of the maneuverability of the probe by using a thin disc-shaped camera with small overall size. The system was developed in a collaboration among the University of Virginia, SurgicEye, the Thomas Jefferson National Accelerator Facility (Jefferson Lab, Newport News, VA), and Dilon Technologies (Newport News, VA). Here, we describe laboratory tests of the system’s imaging properties, and results of a feasibility study using the investigational camera fhSPECT system for preoperative SLN mapping among melanoma surgical patients.

### Imaging system description

**Fig. 1:**
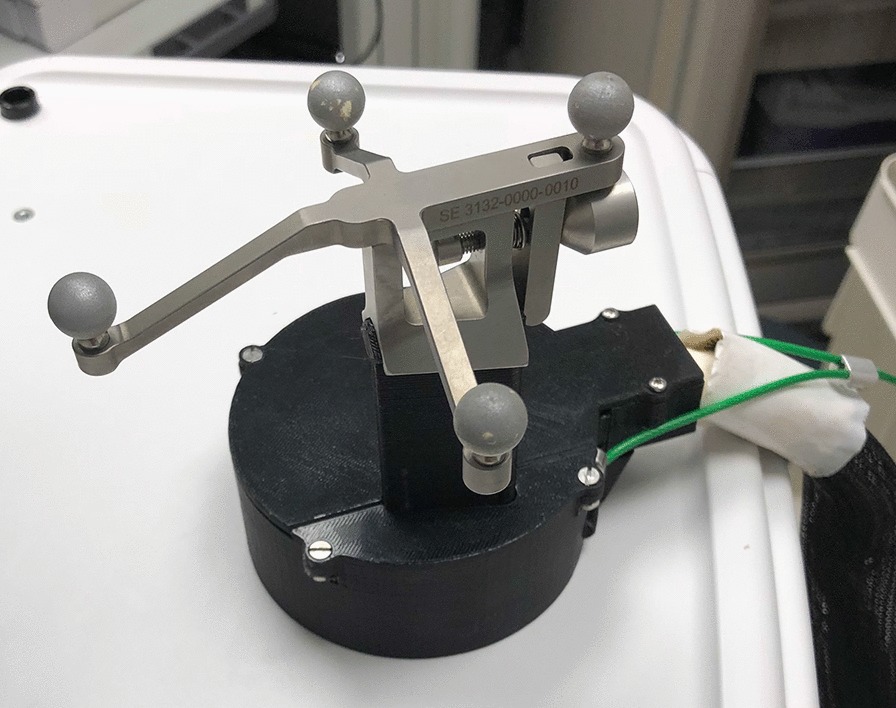
Handheld SiPM gamma camera with four reflecting spheres

The 7-cm-diameter handheld gamma camera (Fig. 1) incorporates a 60-mm-thick pixelated thallium-doped sodium iodide (NaI(TI)) scintillator, an array of 80 silicon photomultipliers (Hamamatsu Photonics model S10362-33-050P) with a pitch of 6 mm, and a custom-built tungsten–polymer composite parallel-hole collimator with hole size 0.6 mm and hole length 5.5 mm [28]. The central region of the NaI(Tl) crystal is pixelated to form a 25 × 25 array of 2.25 mm × 2.25 mm crystals with 2.5 mm pitch. The camera, designed and built at Jefferson Lab, is housed in a cylinder of CMW-1000 machinable tungsten with an interior layer of black Delrin® (polyoxymethylene). This interior layer shields the SiPMs from ambient light and mechanically stabilizes the camera’s electronics. The combined mass of the camera and housing together is 1.4 kg, allowing it to be scanned by hand without mechanical support. The camera’s design and performance with a single-crystal lanthanum bromide scintillator has been described previously [28].

**Fig. 2:**
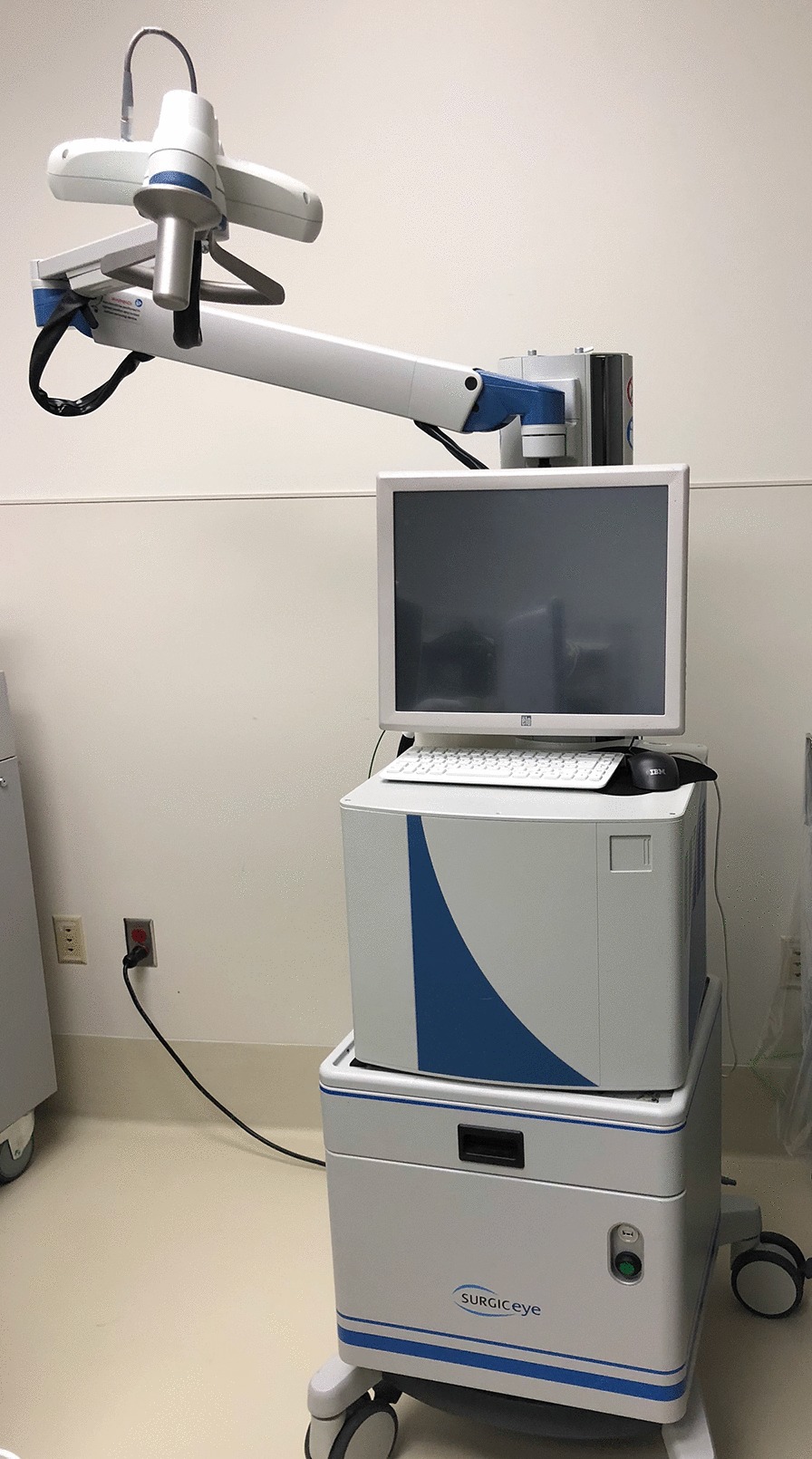
The investigational camera declipse®SPECT system. The SiPM camera is mounted in a holder behind the monitor when not in use

For 3D imaging, the rear surface of the gamma camera housing was fitted with an array of four reflective spheres (Fig. 1) so that IR light from the freehand SPECT overhead tracking system can be used to determine camera location and orientation (Fig. 2). A similar array containing three reflective spheres is placed on the surface of the subject being scanned in order to provide a fixed reference to the subject surface.

During image acquisition, the handheld camera is first swept in a non-imaging fashion over a broad region of the subject’s surface with the goal of identifying areas producing relatively high counting rates. Once high-count regions are identified, a more systematic raster scan is performed in imaging mode to image a cubic volume approximately 1000 cm^3^ (10 cm on each side). Ideally, during the 3D scan the camera is moved to obtain projection images in two or preferably three mutually perpendicular planes. In many cases, three precisely orthogonal camera orientations are impossible because of the node location relative to the surface topography of the subject. However, even in these cases useful 3D images of SLNs can be obtained with nearly isotropic spatial resolution by changing the camera orientation as far as possible during the scan. The total scan time is typically 90 s, at which point the declipse®SPECT software performs image reconstruction. Quantitative data on the depth and intensity of detected focal activity are displayed on a real-time video image of the patient surface.

## Methods:

### Laboratory characterization of imaging properties

#### Energy resolution

A low scatter source comprising a thin layer of [^99m^Tc]pertechnetate solution in a thin plastic petri dish was used to acquire a pulse height histogram. The full width at half maximum of the 140 keV peak was measured to quantify the energy resolution of the handheld SiPM gamma camera.

#### Spatial resolution

To characterize the 2D spatial resolution of the handheld camera, thin line sources were created with [^99m^Tc]pertechnetate in capillary tubes (Kimble 71,900–50 μL, 1 mm inner diameter). The line sources were imaged at 10-mm source-to-collimator intervals beginning at the surface of the collimator until a distance of 100 mm was reached. At each location, the capillary was oriented at a small angle with respect to the crystal matrix so that the width of its image could be averaged over multiple offsets of the capillary from the crystal centers [29].

The reconstructed 3D spatial resolution was measured by placing a drop of [^99m^Tc]pertechnetate solution in the tip of an Eppendorf tube. The source was scanned in air using a source-to-camera separation of ~ 10 cm. The spatial resolution was measured in three perpendicular dimensions by extracting the central slice from the reconstructed volume.

#### Sensitivity

In both 2D and 3D, the sensitivity was defined as the ratio of total number of image counts per second and the source activity. Source size was chosen so that the entire source image fit within the field of view of the handheld SiPM gamma camera.

The 2D sensitivity of the gamma camera was experimentally determined according to the standards of the National Electrical Manufacturers Association (NEMA NU 1-2012 ‘Performance Measurements of Gamma Cameras’). A thin layer of [^99m^Tc]pertechnetate in a 10-mm-diameter petri dish was imaged with a source-to-collimator separation of 10 cm.

The 3D sensitivity of the imaging system was evaluated by imaging Eppendorf tubes with activities in the range of 0.132 to 224 MBq (3.59 to 6060 μCi). A source to collimator separation of approximately 30 cm was used during scanning.

#### Node localization accuracy

After scanning is completed and 3D image reconstruction has been performed, the investigational handheld camera fhSPECT system interactively displays the separation between the gamma camera and regions of focal radioactivity, such as SLNs. If the camera is placed face down on the patient’s surface, this provides a measurement of node depth. A series of tests were performed to determine the accuracy of the localization measurement reported by the imaging system. Such information is useful in surgical planning of optimal paths to nodes and is not readily available with non-imaging gamma probes.

Eight nodes were simulated using small spheres filled with activities ranging from 18 to 52 µCi (average of 26 µCi or 0.96 MBq). Following each node scan, the input surface of the camera was placed in contact with the node and then at a distance of 35 mm.

**Fig. 3:**
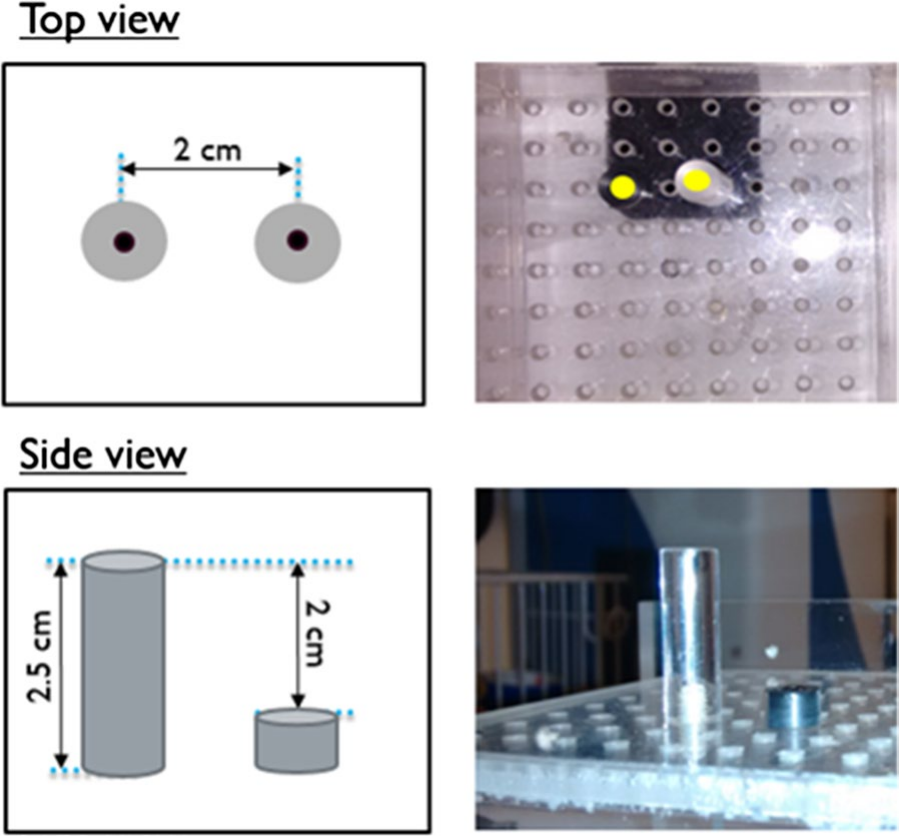
Schematic diagram and photographs of phantom used to evaluate source localization accuracy. The point sources are at the top surfaces of the two acrylic posts

The accuracy of the system’s ability to measure the separation between nodes was evaluated using a phantom containing two point sources at known separations. Figure 3 shows schematic diagrams and photographs of the phantom, which contains two acrylic posts of different heights, with a center-to-center spacing of 20 mm. A drop of [^99m^Tc]pertechnetate solution was added to the small well in the center of each post’s top surface. The phantom was scanned, and then the camera was placed at multiple locations ranging from ~ 5 cm to 20 cm from the sources. The reported source-to-camera separations were used to calculate the inter-source separation in the X, Y, and Z dimensions compared to the known values (20 mm, 0 mm, and 20 mm).

### Human feasibility study

The investigational handheld camera fhSPECT system was tested in a pilot study approved by the Health Sciences Research Institutional Review Board (HSR-IRB, approval #18883) of the University of Virginia. Eligible subjects were surgical melanoma patients of CLK who were scheduled for intraoperative SLNB. The study enrolled seven subjects, the last five of which were successfully scanned. The first two subjects enrolled were not scanned due to software problems in the camera fhSPECT system that were subsequently fixed. All subjects provided informed written consent to participate. Subjects were scanned during their waiting time in their normal preoperative suite. Aside from this, subjects received only procedures that were part of their standard care at the University of Virginia Health System, including [^99m^Tc]sulfur colloid injection, lymphoscintigraphy imaging, and intraoperative SLN localization using a non-imaging probe. No additional radiotracer beyond that injected for routine clinical care was used for the investigational camera fhSPECT scans; thus, subjects were not exposed to any additional ionizing radiation in order to participate in this study. Surgeons viewed the preoperative lymphoscintigraphy images prior to surgery per standard clinical practice. However, they were kept blinded to the results of the preoperative fhSPECT scans (both probe fhSPECT and camera fhSPECT) so that surgical procedure was not affected by the study.

**Fig. 4:**
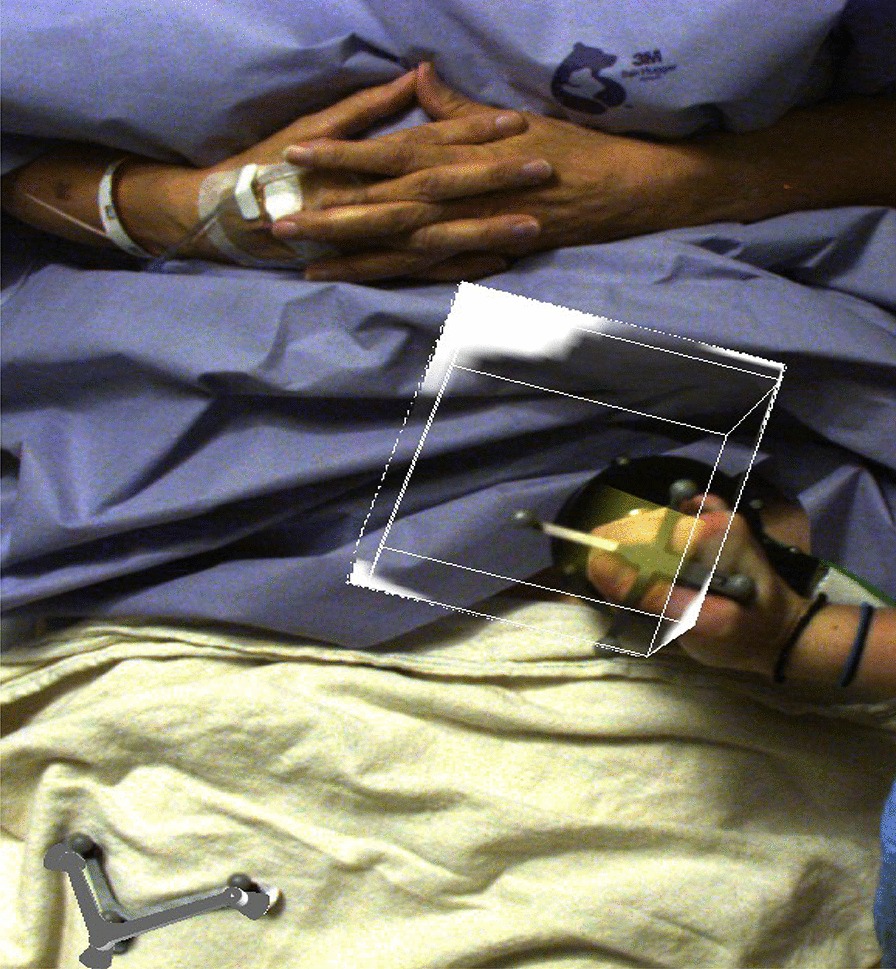
Subject being scanned with the camera fhSPECT system. The box indicates the volume that will be reconstructed

For each subject, camera scanning was performed for 90 s per region imaged. For each scan, the location of the ~ 10 cm × 10 cm × 10 cm volume of view (VoV) was determined by a count rate survey using the handheld camera to identify regions of focal uptake. During the subsequent 3D scan, information on the specific location, depth, size, and activity of each node was displayed on the system monitor. At the end of the scan, the information was overlaid on a video image of the subject (Fig. 4). In some incidences, a second camera fhSPECT or a second probe fhSPECT scan of a given basin was performed when the first scan showed one or more SLNs lying near the periphery of the scanned VoV. Thus, the second scan was performed in order to better center the SLNs in the VoV.

For three subjects, 3D scans using a non-imaging probe coupled to the declipse®SPECT system were performed following the 3D camera fhSPECT scans using the SiPM camera. The protocol for the 3D scans using the non-imaging probe was the same as that used with the commercially available, FDA-approved declipse®SPECT system. Probe fhSPECT scans were not done in two cases where doing so would have delayed the start of surgery. When they were possible, probe fhSPECT scans were conducted of the same lymphatic basins as were imaged using camera fhSPECT so that the images could be compared.

For each subject, the total number of SLNs detected by each modality (lymphoscintigraphy, camera fhSPECT, probe fhSPECT, and intraoperative non-imaging probe) was determined. In cases where more than one camera fhSPECT or more than one probe fhSPECT scan was performed on the same nodal basin, only the scan that was best centered on the basin was used when determining the total number of imaged SLNs. Other scans were not considered when counting SLNs to avoid the possibility of counting a single node twice.

In order to be classified as a SLN in the 3D modalities, a volume of focal uptake (a hot spot) had to span at least five consecutive image slices. This criterion was established experimentally to discriminate node hot spots from background noise counts in the images. In experiments using node-size acrylic shells filled with varying activities (0.015–0.56 MBq, or 0.4–15 µCi) of ^99m^Tc solution, reconstructed images of the node phantoms consistently spanned at least five image slices, while noise-induced hot spots rarely spanned more than one or two image slices. This remained true even for node phantom activities of less than one µCi (< 0.037 MBq).

During surgery, the ‘10% rule,’ which is widely utilized in surgical SLNB for deciding which nodes should be excised, was applied. According to this criterion, only nodes with radiation activities greater than or equal to 10% of that of the hottest node (as determined by in vivo counting rate measurement using the non-imaging gamma probe) are classified as sentinel. Node activity (i.e., amount of tracer in the node) is not indicative of the number of malignant cells, if any, that are trapped in the node. However, the nodes with the most direct draining paths from the cancer are the most likely to harbor malignant cells from the tumor. Since those direct drainage nodes are also the ones likely to accumulate the greatest amount of colloidal radiotracer, the 10% rule is employed in SLNB in an effort to evaluate only the nodes most likely to be positive for malignancy and avoid unnecessary removal of higher echelon nodes. The threshold value of 10% remains somewhat controversial, with some studies suggesting that it may result in removal of a larger number of nodes than necessary [30, 31] and others determining that higher threshold values result in an unacceptable increase in the false negative rate (omission of nodes actually positive for metastasis) [32]. Nevertheless, to be consistent with surgical practice during this study, the 10% rule was also applied to all nodes identified in the 3D scans as follows. In each volumetric image, the total activity of each node was determined by summing the values of all voxels within the node in each slice through which the node extended. Nodes whose total activity was less than 10% of that of the brightest node in the nodal basin were not considered sentinel and were discarded from the data. Nodes in lymphoscintigraphy images were counted if they visually appeared to be at least 10% as bright as the brightest node. Nodes detected intraoperatively using the non-imaging probe were subjected to the 10% rule and counted as SLNs if they were excised. Side-by-side comparisons were made of nodes visible in lymphoscintigraphy, probe fhSPECT, and camera fhSPECT images for qualitative assurance that the same nodal basins were being compared.

## Results

### Laboratory characterization of imaging properties

#### Energy resolution:

Averaged over seven trials, the energy resolution was measured to be 21.5 ± 1.7% (mean ± 95% confidence interval) at 140 keV.

#### Spatial resolution

**Fig. 5.**
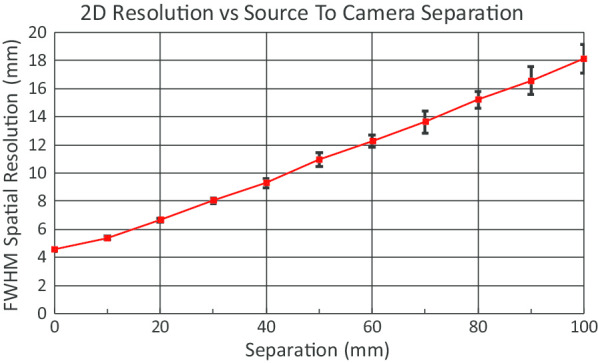
2D spatial resolution of NaI(Tl)–SiPM gamma camera. Error bars indicate the 95% confidence intervals derived from six trials. The image pixel size is 2.5 mm

2D: The phase-averaged FWHM of the width of the capillary image in each 2D projection image is plotted versus changing capillary-to-collimator separation in Fig. 5. The average of seven trials is shown.

3D: Averaged over three scans, the FWHM of the image of the point source was 11.9 ± 2.5 mm, 13.3 ± 0.3 mm, and 14.2 ± 1.8 mm in the coronal, sagittal, and axial planes, respectively. These three planes were defined in terms of a supine surgical patient.

#### Sensitivity

**Fig. 6:**
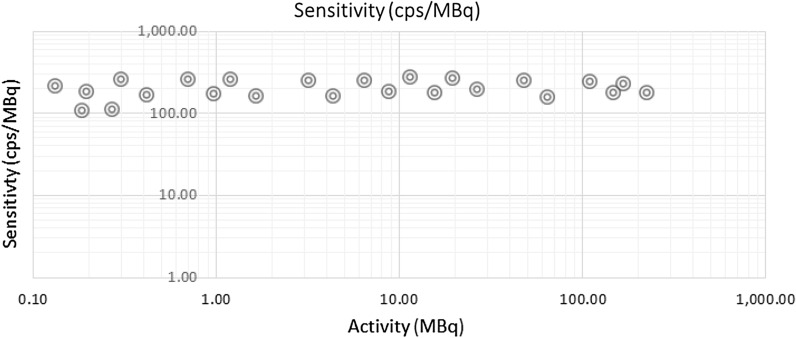
System photon sensitivity for 3D imaging over a range of source activity

The 2D sensitivity of the SiPM gamma camera was found to be 171 ± 6.2 cps/MBq. 3D sensitivity is shown plotted versus source activity in Fig. 6. The results show that the 3D sensitivity is uniform in this range of activity with a mean sensitivity of 203 ± 19.5 cps/MBq. The small difference between the 2D and 3D sensitivity is attributable to the use of a slightly more narrow energy window in 2D imaging compared to a wider energy window used for 3D scanning.

#### Node localization accuracy

**Fig. 7:**
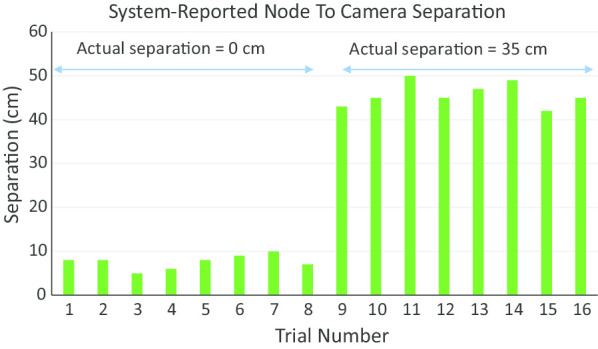
Camera-to-node separation reported by the imaging system for eight simulated nodes. For each node, two actual separations (0 cm and 35 cm) were tested. The error in the reported distances was 9.2 mm with a standard deviation of 2.7 mm. Taking into account the 9 mm combined thickness of the collimator and camera housing, this corresponds to a true error of 0.2 ± 2.7 mm

The bar plot of Fig. 7 compares the known separations between the camera and the eight simulated nodes to those reported by the imaging system. For the 16 trials (eight nodes and two post-scan camera positions each), the average error in the reported camera-to-node separation was 9.2 mm with a standard deviation of 2.7 mm. However, the separation reported by the system is actually that between the node and the input surface of the NaI(Tl) scintillator. That surface is separated from the camera’s outer surface by the thickness of the camera housing. Taking into account the combined 9 mm thickness of the collimator and camera housing, this corresponds to a true error of 0.2 ± 2.7 mm.

Averaged over all camera-to-phantom distances tested, the mean absolute error in the inter-node separation, computed from system-reported node locations, was 1.2 ± 0.34 mm (95% confidence interval).

### Human feasibility study

**Fig. 8:**
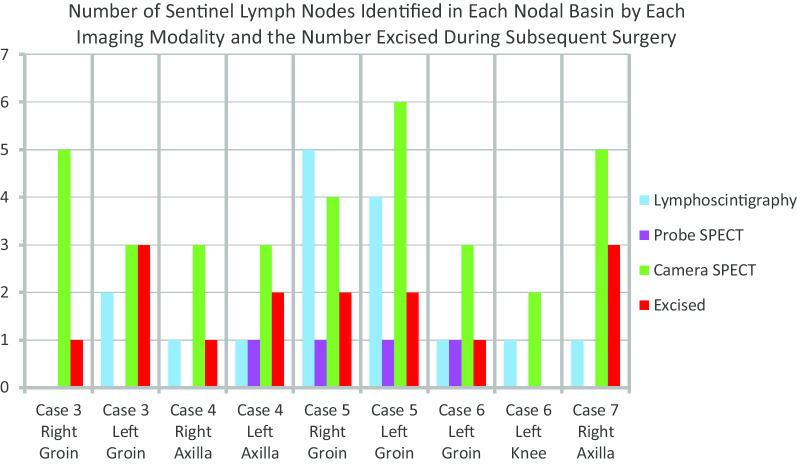
Bar graph comparing, for each of nine lymphatic basins, the number of SLNs detected by each of the three imaging modalities tested and the number excised

A breakdown of the human study results in terms of the numbers of SLNs imaged and excised, along with the lymphatic basins in which they were located, is presented in Table 1 and Fig. 8. Fifteen total nodes were excised from the five subjects. Of those, only one node (left groin, subject #5) was positive for malignancy.

**Table 1:** Number and location of SLNs imaged by lymphoscintigraphy, preoperative probe fhSPECT, and preoperative camera fhSPECT, and number and location of SLNs subsequently excised during surgery

Case	Melanoma site	Lympho-scintigraphy	Probe fhSPECT	Camera fhSPECT	Excised
3	Right lower abdomen	2 Left groin 0 Right groin	No scan No scan	3 Left groin 5 Right groin	3 Left groin 1 Right groin
4	Midline epigastrium	1 Left axilla 1 Right axilla	1 Left axilla No scan	4 Left axilla 3 Right axilla	2 Left axilla 1 Right axilla
5	Lower back	4 Left groin 5 Right groin	1 Left groin 1 Right groin	2 Left groin 4 Right groin	2 Left groin 2 Right groin
6	Left heel	1 Left knee 1 left Groin	0 Left knee 1 Left groin	2 Left knee 3 Left groin	0 Left knee 1 Left groin
7	Right upper back	1 Right axilla	No scan	5 Right axilla	3 Right axilla

Table 1 shows that for a number of basins (Case 3, left and right groins; Case 4, left axilla; and Case 7, right axilla) lymphoscintigraphy identified fewer SLNs than were ultimately excised, suggesting false negatives via lymphoscintigraphy. However, for each of those basins the number of nodes identified by camera fhSPECT was equal to or greater than the number ultimately excised.

Probe fhSPECT scans were performed in Cases 4–6, but were precluded by insufficient pre-op time in Cases 3 and 7. For the same reason, the right axilla in Case 4 was not scanned. In the left axilla of Case 4, probe fhSPECT detected one fewer SLN than was excised. The same was true for both basins in Case 5. In Case 6, the number of SLNs detected by probe fhSPECT matched the number excised in both basins.

For seven of the nine lymphatic basins imaged, the number of nodes identified by the investigational handheld camera fhSPECT system was either greater than or equal to the number visualized via lymphoscintigraphy. In Case 5, the number of nodes detected by lymphoscintigraphy exceeded that detected by camera fhSPECT in both the left and right axillary basins.

Three subjects (Cases 4, 5, and 6) were scanned with both probe fhSPECT and camera fhSPECT. For each nodal basin scanned in these three subjects, camera fhSPECT was able to visualize a greater number of nodes than probe fhSPECT. This difference could be attributable to the increased tomographic sampling available with the multi-detector element SiPM camera compared to the single-detector element probe.

There were no nodal basins for which the number of SLNs detected via camera fhSPECT was fewer than the number ultimately excised. However, in seven basins the total number of hot spots imaged via preoperative 3D scanning was greater than the number of nodes excised. Unfortunately, there was no way for nodes that were detected by 3D imaging, but not identified for excision conventionally, to be investigated during surgery or histologically, because the IRB-approved study protocol stipulated that node localization and excision were to be performed according to the current standard of care (guided only by lymphoscintigraphy and the non-imaging probe). Thus, the surgical team was blinded to the results of the 3D scans.

Nodes detected via imaging but not excised could fall into one of several categories. They could be nodes that were simply not detected by the non-imaging probe. Alternatively, they could be nodes that were detected by the probe but were not excised because their activity as measured in vivo with the probe was below the 10% threshold. It is possible for deeper lying nodes with in vivo activities below the 10% exclusion threshold to nevertheless have image-determined activities above the threshold because the attenuation correction algorithms built into the declipse®SPECT iterative reconstruction software can partially compensate for attenuation of node-emitted gamma rays by overlying tissue. In these cases, the activity determined from the 3D scan may be more accurate than that determined via the non-imaging probe. It is also possible for a node to be detected by the probe and pass the 10% criterion, but be intentionally not excised because of surgical considerations such as a risk of complications associated with the required surgical path.

In most institutions, radiotracer injection and lymphoscintigraphy are performed in the nuclear medicine clinic rather than the surgical preoperative suite. Inter-site scheduling offsets and patient transfer times often dictate that injection/lymphoscintigraphy occur on the day prior to surgery, especially for patients scheduled for morning surgery. Studies comparing early (1-day) and delayed (2-day) SLNB protocols in breast cancer have demonstrated that the patterns of distribution of the sulfur colloid radiotracer in the lymph nodes and the results of SLNB are virtually identical for the early and delayed protocols [33, 34]. However, the delayed protocol results in significant radioactive decay of the ^99m^Tc (half-life = 6.0 h) lymphatic tracer during the time interval between tracer injection and surgery and thus very low (~ 2 MBq) SLN activity. Therefore, it was important to evaluate the ability of the investigational fhSPECT system to visualize SLNs with very low activity.

Table 2 shows, for each of the five cases reported here, the elapsed time between injection and lymphoscintigraphy (always 15-min post-injection at our institution) and between injection and 3D scanning (probe fhSPECT and/or camera fhSPECT), along with the fractional reduction in the SLN activity. In three of the five cases, less than 15% of the SLN activity at the time of lymphoscintigraphy remained when the 3D scans were performed. In this study, for all but two basins (the left and right groins in Case 5), the number of SLNs detected by camera fhSPECT was always equal to or greater than the number of SLNs detected by lymphoscintigraphy. The fact that this was true even when less than 15% of the activity remained during the 3D scan demonstrates the high sensitivity of camera fhSPECT.

**Table 2:** SLN radioactive decay for each of the five cases in this study. In the three cases shaded, less than 15% of the SLN activity present at lymphoscintigraphy remained at probe fhSPECT or camera fhSPECT imaging

Case #	Injected activity (MBq)	Injection—LS time (hrs)	Injection—3D scanning time (hrs)	Fraction of injected activity remaining at LS	Fraction of injected activity remaining at 3D scans
3	18.5	0.25	19.7	0.97	0.10
4	20.4	0.25	3.1	0.97	0.70
5	18.9	0.25	18.4	0.97	0.12
6	20.4	0.25	22.7	0.97	0.07
7	18.5	0.25	4.4	0.97	0.60

The two 3D scans (camera fhSPECT and probe fhSPECT) were performed with no lapsed time between them, so their injection-to-scan time was identical). LS = lymphoscintigraphy; 3D scans = camera fhSPECT and (when performed) probe fhSPECT

## Discussion and conclusions

To date, two handheld gamma cameras have been integrated with within the 3D image acquisition framework of the declipse®SPECT tracking system and image reconstruction software. One is the developmental SiPM-based handheld gamma camera described here and previously in the context of 2D intraoperative imaging [28], and the other is the commercially available mobile gamma camera (CrystalCam). Table 3 compares the primary physical characteristics of the two cameras.

**Table 3:** Comparison between the SiPM-based handheld gamma camera of this study and the CZT-based CrystalCam (Crystal Photonics), both of which have been integrated with declipse®SPECT

	SiPM camera	CrystalCam
Detector material	NaI(Tl)	CZT
Active area shape	square	square
FOV (mm)	63 × 63	40 × 40
Active area (sq. mm)	3906	1600
Number of detector elements	625	256
Detector element pitch (mm)	2.5	2.5
Energy resolution at 140 keV	22%	< 7%
Overall physical dimensions (mm)	75 (dia) × 41 (H)	60 × 60 × 160 (H)
Mass (kg)	1.4	0.8

In a human study including 10 subjects undergoing SLN biopsy for breast cancer, a CZT-based camera fhSPECT system described by Engelen et al. identified 11 of the 12 nodes detected via lymphoscintigraphy [27]. The camera fhSPECT system successfully provided surgical navigation to all nodes it visualized, and overcame some of the sensitivity limitations of the non-imaging gamma probe.

The same gamma camera fhSPECT system was later combined with indocyanine green (ICG) fluorescence imaging in a hybrid system [12]. Using a dual-modality ICG-[^99m^Tc]nanocolloid tracer developed in the van Leeuwen laboratory [35, 36], a study including eight head and neck melanoma subjects was performed. Intraoperative camera fhSPECT was followed by 2D fluorescence imaging to confirm the exact locations of the SLNs. The hybrid system successfully visualized 14 of the 15 preoperatively identified SLNs.

**Fig. 9:**
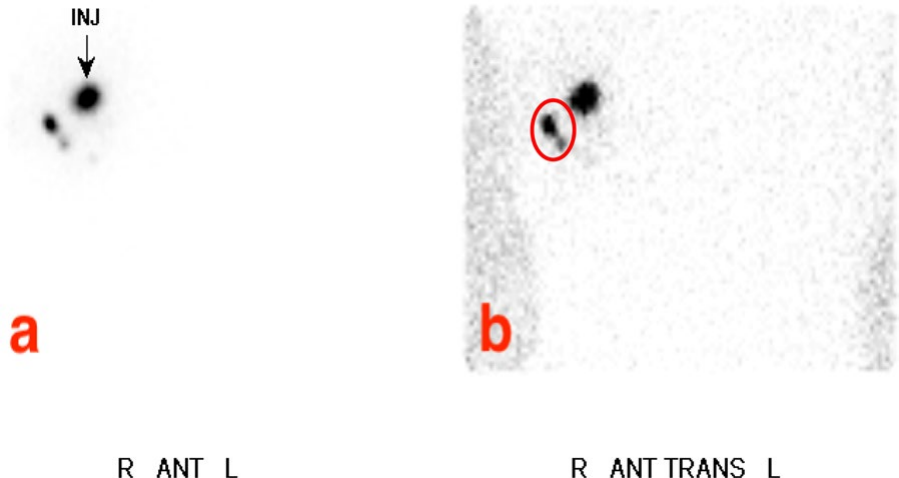
Preoperative lymphoscintigraphy images, Case 7

**Fig. 10:**
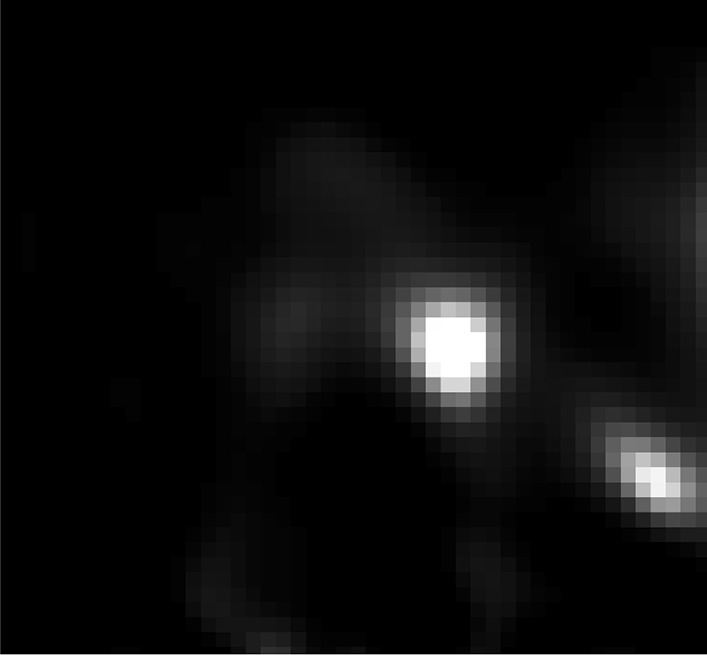
Camera fhSPECT slice, Case 7, corresponding to the red-circled portion of Fig. 9b

Spatial resolution impacts the ability of an imaging system to distinguish between two separate sources of activity (e.g., two closely spaced SLNs) as well as its ability to determine their relative activities. The experimentally determined spatial resolution of the handheld camera fhSPECT system is approximately 12—14 mm, depending on the particular scanning geometry and the source-to-camera separation during the scan. Therefore, it is possible that two SLNs spaced less than 1 to 1.5 cm apart may be counted as one. The same is true for handheld probe fhSPECT and lymphoscintigraphy, where spatial resolution is even lower in each case. Figures 9 and 10 show an example of how the superior spatial resolution of camera fhSPECT was able to better separate two closely spaced SLNs, compared to lymphoscintigraphy. In case 7, lymphoscintigraphy only identified one node in the right axilla, but camera fhSPECT was able to separate what appeared to be one node with a tail into two separate nodes (Figs. 9 and 10).

In SLN mapping, 3D imaging has the inherent advantage compared to 2D imaging that it permits the surgeon’s direction of observation to be chosen post-image acquisition. For example, the ^99m^Tc-labeled colloidal tracer is typically injected near to the site of the known cancer(s). Since a significant amount of activity remains local to the injection site and only a few percent drains to any given lymph node, the residual activity at the injection site is typically much higher than that of individual SLNs and obscuration of nodes in lymphatic basins lying near the melanoma lesion(s) is common. In those cases, detection of SLNs that would satisfy the 10% rule but are near to the lesion can be difficult because of complete or partial superposition of the signals from the injection site and the node in the 2D image of a mobile gamma camera, or within the FOV of a non-imaging probe. A potential advantage of 3D imaging techniques is that the reconstructed volume can easily be reformatted to obtain slices with optimal viewing perspectives permitting close SLNs to be differentiated from the residual injection site activity [37].

Due to the high node detection sensitivity of the investigational fhSPECT system, there were a number of basins in this study where more nodes were detected via camera fhSPECT than were ultimately excised. Possible reasons for this were enumerated in Sect. 3.1. While ideally in SLNB only nodes needed for definitive assessment of lymphatic metastasis would be excised, identification of this unique minimum necessary set via imaging is not feasible. For example, the extent (e.g., involvement of first echelon versus higher echelon nodes) and pattern of tracer drainage and retention in the nodes depends on variables such as the location of the primary tumor and tracer particle size. It is possible that some of the imaged nodes in this study, whether excised or not, included higher echelon nodes. The 10% rule [30–32] constitutes a standard clinical guideline to identify sentinel nodes and was used in this study as part of the standard of surgical care at our institution. Under its guidance, even nodes whose status as higher echelon can be inferred from anatomical location are considered SLNs if their activity is ≥ 10% of that of the hottest node. To limit the number of excised nodes through the development of an imaging system with a built-in lowered sensitivity threshold would be suboptimal as it may result in the surgeon incompletely scanning a nodal basin and missing a true SLN. Optimally, decision on which nodes are excised is left to the surgeon after being presented with all the information available regarding the locations and uptake of the nodes.

Although the results of this feasibility study provide encouragement that fhSPECT incorporating a SiPM-based gamma camera is an effective technique for assessment in 3D of SLN number and locations, the system tested here could be improved in several ways. First, the SiPMs in our camera are early generation devices and exhibit large gain variation with changing ambient temperature. Such temperature sensitivity precludes the use of normal size energy windows for scatter rejection because of the risk of rejecting an unacceptably large fraction of primary (non-scattered) gamma rays as the gain varies. Newer generations of SiPMs have much lower temperature sensitivity compared to earlier ones. Second, the camera’s tungsten housing covers all sides of the camera except the input surface, and its weight makes it somewhat tiring to scan, even for the relatively short 90-s scans used in this study. Housing designs could be explored in which the camera’s rear surface is unshielded or less shielded, thereby greatly reducing the camera weight. Such designs may be sufficient for applications such as SLN mapping in which the tracer concentration tends to lie almost entirely within a limited region that is always in front of the camera. Third, the parallel-hole collimator used in these studies was originally designed for intraoperative 2D scintigraphy. Collimator optimization studies are needed to determine the physical parameters providing the best trade-off between spatial resolution and sensitivity in the context of fhSPECT where, for example, spatial resolution recovery algorithms during image reconstruction might permit higher collimator sensitivity and shorter scan time while still providing adequate reconstructed spatial resolution.

Camera fhSPECT is a potential means for efficiently obtaining real-time 3D activity distribution maps in applications such as image-guided percutaneous biopsy, and surgical SLNB or radioguided tumor excision. Preoperative use of a handheld camera fhSPECT imaging system could improve preoperative planning for sentinel lymph node biopsy by providing information on the distribution, extent, and depth of the colloidal radiotracer immediately prior to surgery. Intraoperative use of camera fhSPECT could permit real-time assessment of residual activity following node excision. A future human study, such as one designed so that handheld fhSPECT images are made available to the surgeons near the end of the surgical procedure, is needed to better measure the specificity of this system intraoperatively.

## Data Availability

The datasets used and/or analyzed during the current study are available from the corresponding author on reasonable request.

## Abbreviations

SPECT: Single-photon emission computed tomography
fhSPECT: Freehand SPECT
SLN: Sentinel lymph node
SLNB: Sentinel lymph node biopsy
NEMA: National Electrical Manufacturers Association
FOV: Field of view
VoV: Volume of view
µCi: MicroCurie
MBq: MegaBecquerel
SiPM: Silicon photomultiplier
